# New Dimensional Staging of Bisphosphonate-Related Osteonecrosis of the Jaw Allowing a Guided Surgical Treatment Protocol: Long-Term Follow-Up of 266 Lesions in Neoplastic and Osteoporotic Patients from the University of Bari

**DOI:** 10.1155/2014/935657

**Published:** 2014-06-05

**Authors:** Simonetta Franco, Simona Miccoli, Luisa Limongelli, Angela Tempesta, Giorgio Favia, Eugenio Maiorano, Gianfranco Favia

**Affiliations:** ^1^Department of Interdisciplinary Medicine, Odontostomatology Unit, Faculty of Medicine, University of Bari Aldo Moro, Piazza G. Cesare 11, 70124 Bari, Italy; ^2^Plastic, Reconstructive and Aesthetic Surgery Unit, Campus Bio-Medico University, Via Alvaro del Portillo 21, 00128 Rome, Italy; ^3^Department of Emergency and Organ Transplantation, Pathological Anatomy Unit, Faculty of Medicine, University of Bari Aldo Moro, Piazza G. Cesare 11, 70124 Bari, Italy

## Abstract

Bisphosphonate-related osteonecrosis of the jaw (BRONJ) is the most serious side effect in patients receiving bisphosphonates (BPs) for neoplastic disease and osteoporosis. The aim of this study is to propose a new dimensional stage classification, guiding the surgical treatment of BRONJ patients, and to evaluate the success rate of this new management. From 2004 to 2013, 203 neoplastic and osteoporotic patients with 266 BRONJ lesions were referred to the Odontostomatology Unit of the University of Bari. All patients underwent surgery after suspension of BPs therapy and antibiotic treatment. The surgical procedure was complemented by piezosurgery and followed by the application of hyaluronate and amino acids. The new dimensional staging suggests the choice of the surgical approach, and allows the prediction of postoperative complications and soft and hard tissues healing time, guiding the surgical treatment protocol. This protocol could be a successful management strategy for BRONJ, considering the low recurrences rate and the good stabilisation of the surgical sites observed after a long-term follow-up.

## 1. Introduction

Bisphosphonates (BPs) are synthetic drugs analogues of inorganic pyrophosphate that can be divided into two groups, nitrogen-containing and non-nitrogen-containing BPs, with different mechanisms of action on osteoclasts [1, 2]. These compounds were originally licensed for the management of skeletal complications of malignancy, including advanced breast cancer and multiple myeloma, but now they are also the drugs of choice in the management of other bone disorders including osteoporosis, cancer-induced hypercalcaemia, Paget's disease, osteogenesis imperfecta [3–5], primary and secondary hyperparathyroidism, and other conditions that feature bone fragility [1]. The most serious side effect of BPs therapy is the bisphosphonate-related osteonecrosis of jaw (BRONJ), firstly described in 2003 by Marx [6]. BPs decrease both bone reabsorption and formation, leading to increased bone fragility and fractures caused by inability to replace old bone by young bone and to repair Microtracks [7]. According to the most widely used definition, given by the American Association of Oral and Maxillofacial Surgeons (AAOMS) and modified by Colella et al., BRONJ is the presence of exposed or otherwise necrotic bone for at least 8 weeks in patients with exposure to BPs and no history of radiotherapy to the jaw [6, 8, 9]. BRONJ can occur in patients receiving BPs therapy and appears to be associated with previous dental traumatic injury; however, spontaneous occurrence has also been observed [6, 10–13]. Most of the incidences of BRONJ have been reported as a result of intravenous administration of high doses of aminobisphosphonates [14, 15], ranging from 0.8% to 12% [16, 17], whereas association of BRONJ and non-nitrogen BP is very rare [18], ranging from 0.01 to 0.34% [16, 17]. The risk of BRONJ development rises in the presence of long duration of BPs exposure, concomitant treatment with corticosteroids [19–21], chemotherapies [22, 23], antiangiogenic drugs [24–26], and hormone therapy or in the presence of patient comorbidities such as immunodeficiency, diabetes mellitus, obesity, hypercholesterolemia, and parodontopathies.

The existing BRONJ staging systems are numerous, and most of those systems are based on clinical findings: Ruggiero et al. in 2006 proposed a clinical staging system which recognizes three different clinical levels based on signs and symptoms [27]; then, the American Association of Oral and Maxillofacial Surgeons (AAOMS) in 2009 implemented his staging with Stage 0 [16]. Marx in 2007 [28] was the only one who divided the stages into substages according to the lesions size; and Bedogni et al. in 2012 proposed a combined clinical and radiological staging system to divide BRONJ patients into groups on the base of the radiological findings [29] (Table 1).

All of these staging systems are useful from a clinical and diagnostic point of view, but no one is surgical oriented, so no one can guide the surgeon in the management of BRONJ patients.

There are still controversies also about the adequate treatment of patients affected by BRONJ with regard to BPs discontinuation, medical therapy, surgery, or other therapies (hyperbaric oxygen therapy, ozone therapy, and laser therapy).

The rationale for BPs discontinuation is the interruption of their effects on the oral tissues, but no real good effect on BRONJ treatment connected with BPs suspension has been reported in the literature [30].

The general medical therapy consists of the combination of amoxicillin (2 g/day) and metronidazole (1.5 g/day) for at least two weeks to cover most bacteria isolated [16]. The main limitation of this therapy is the temporary clinical results, followed by a relapse of infections and symptoms after some weeks [31].

In many recent studies, surgical debridement or marginal resection, in combination with antibiotic therapy, presented better results than just medical treatment. AAOMS recommendations regarding surgery were limited just to Stage III, but several studies showed optimum results of surgical procedures also in Stage I and Stage II [32]. In last years, also other noninvasive therapies were proposed, such as hyperbaric oxygen therapy, ozone therapy, which can improve the vascular flow, and laser therapy, which can be used for biostimulation (low-level laser therapy (LLLT)) or conservative surgery, through bone vaporization by Er:YAG laser, until healthy bone is reached [30]. The aim of this study is to evaluate the outcomes of 266 BRONJ lesions in 145 neoplastic and 58 osteoporotic patients after the surgical management guided by a new dimensional stage classification.

## 2. Materials and Methods

From 2004 to 2013, a total of 203 patients suffering from BRONJ were referred to the Odontostomatology Unit of the University of Bari and were included in this retrospective study. The criterion for inclusion was current or previous bisphosphonate therapy due to osteoporosis or cancer disease. Patients who received radiation therapy in the oral and maxillofacial area, with an estimated overall life expectancy less than 1 year, or in presence of contraindications for general anaesthesia, were excluded from the study. A database record was designed for each included patient, with a detailed history concerning gender, age, primary disease, BPs used, administration, dose and duration of therapy, suspension of the therapy, clinical stage, size, multifocality, comorbidity, site, trigger, symptoms, signs, and recurrences.

The BRONJ lesions were staged according to their size after OPT and CT evaluation, and the surgical approach was different according to the stage (Table 2).

Our treatment protocol consisted of the following steps:

- radiographic evaluation;
- suspension of BPs therapy if systemic conditions permit;
- administration of ceftriaxone and metronidazole;
- surgical debridement or marginal resection according to the stage (Figure 5);
- hyaluronic acid and amino acids application;
- histopathological analysis;
- BPs resumption not before 1 month after surgery;
- clinical and radiological follow-up.

**Figure 1:**
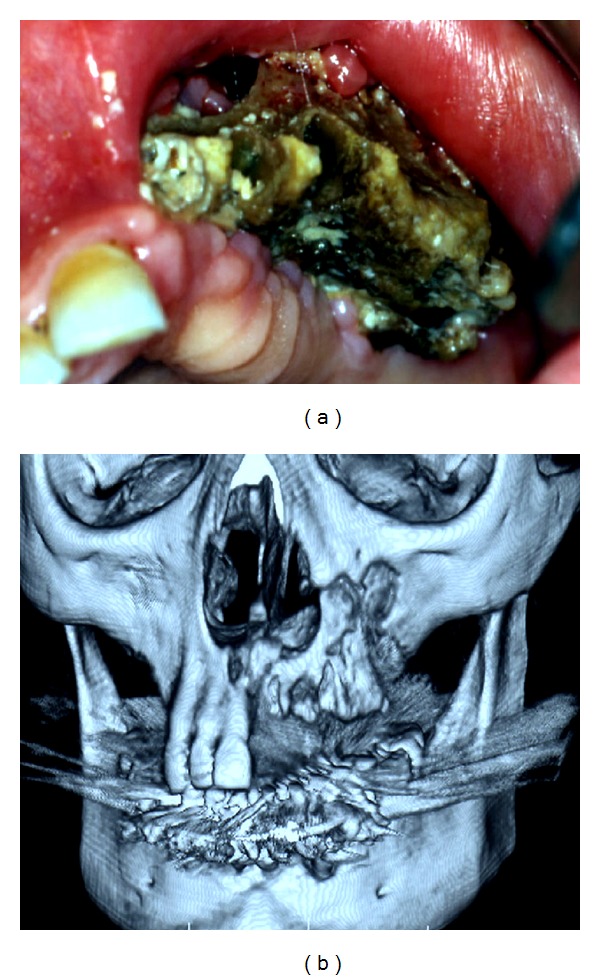
Clinical aspect and multislice spiral CT with 3D reconstruction of Stage III BRONJ involving the maxillary sinus, in a 74-year-old female patient with multiple myeloma, who underwent zoledronic acid therapy.

The radiographic evaluation was made through OPT examination and multislice spiral CT with 3D reconstruction (Figure 4), and all lesions were measured in centimetres to adequate the surgical treatment (Figure 1).

When it was possible, each patient suspended BPs therapy not less than 3 months before surgical procedure, and corticosteroids and chemotherapy were suspended, too, taking into account general conditions of patients and upon consultation with the treating physician and the patient. At least, 3 cycles of antibiotic therapy were administered. Every cycle consisted of a combination of ceftriaxone (1 g once a day i.m.) and metronidazole (500 mg twice a day per os) administered for 8 days with 10 days of interruption after each cycle.

The marginal bone resection included at least 1 cm of vascularized bone tissue extended in depth and in all the sides. The depth of resection was pinpointed by the bleeding evaluation of bone tissues. Noble structures and cortical bone were preserved where it was possible.

Surgery was complemented by using vibrating tips connected to a high power ultrasonic device (piezosurgery) for the osteoplasty of the residual resection margins and with the application of a medical device made of hyaluronic acid and amino acids (glycine, leucine, lysine, and proline).

The same medical device was put on the stitches from the patients (sandwich technique), after wound rinse by saline solution and hydrogen peroxide, at least three times a day until stitches removal. If there was sinus maxillary involvement, the Caldwell-Luc technique was used.

**Figure 2:**
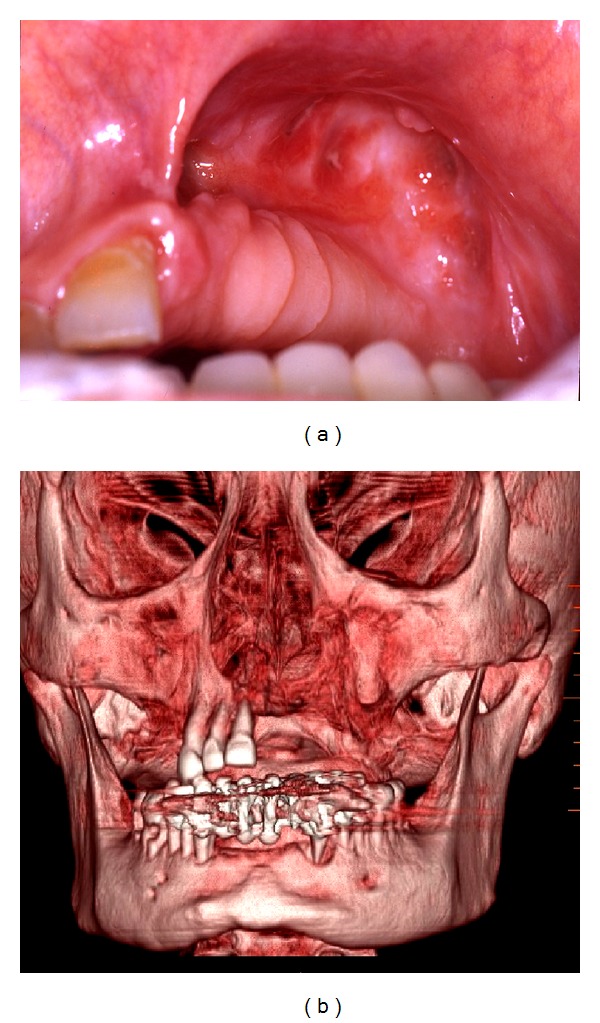
Complete bone and mucosal healing and multislice spiral CT with 3D reconstruction 13 months after surgery and intracavitary application of Aminogam gel.

All the samples were fixed in 10% neutral buffered formalin and sent to the Pathological Anatomy Unit of University of Bari, paraffin embedded, thin sectioned at 3 *μ*m, and stained with haematoxylin-eosin (Figure 6). The histological examination was carried out using Nikon Eclipse E600 microscope (Nikon Corporation, Tokyo, Japan), equipped with Argon and Helio-Neon lasers, emitting at 488 nm and 543 nm wavelengths, which allows both optical and confocal laser scanning microscope (CLSM) analysis. The Nikon EZ C1 software (Nikon Corporation, ver. 2.10, Coord Automatisering) was used for bidimensional image processing. Patients could receive again BPs therapy after the complete soft tissues healing, at least 1 month after surgery. Each patient underwent an accurate clinical follow-up each week in the first month and then clinic-radiographic follow-up at 1, 3, 6, and 12 months after surgery (Figure 2).

In 20 osteoporotic patients, low-level laser therapy (LLLT) was performed for the first time during the surgical intervention directly on the residual vital bone and then three times a week for three weeks on the soft tissues. Each LLLT application was performed with Diode Laser (A2Glaser “Surgery 35”) employed with a fibre of 320 *μ*m, a wavelength of 800 ± 10 nm, and an energy output of 2 Watt. It was used in pulsed mode (on 50 ms/off 50 ms) and in a nonfocused way, at 2 mm from tissues for 1 minute, and repeated for three times.

After the 12-month follow-up, we defined “clinical success” as a treatment able to give a positive result in terms of patient quality of life that could be

1. complete healing without symptoms or clinic-radiographic signs;
2. transition from a higher to a lower stage of BRONJ site according to AAOMS staging (healing improvement);
3. healing with after-effects considering bone, periodontal, or dental deficit after surgery,

whereas we defined “recurrence” as the clinic-radiographic representation of BRONJ in the same site or in adjoining sites within 12 months from the surgery.

Data were entered into a FileMaker Pro Database and analysed using STATA MP11. The association among several variables was tested using the *χ* ^2^ test or Student's *t*-test, where appropriate, and a multiple logistic regression model was applied to evaluate the determinants of multifocality, stages, symptoms, and signs. Odds ratios (OR), 95% confidence intervals (CI), and the value of *Z*-test were calculated, and a *P* value ≤0.05 was chosen for statistical significance.

## 3. Results and Discussion

### 3.1. Sample Characteristics

Out of 203 patients, 75.37% were females; the data confirmed the high prevalence of BRONJ among women in the literature. The age range was 38 to 94 years, with a mean age of 67.8 ± 11.3 years. Among the 203 BRONJ patients, an oncologic diagnosis had been made in 71.43% of cases, whereas the 28.57% of patients received BPs for osteoporosis.

The BP most used was zoledronate, followed by alendronate, clodronate risedronate, ibandronate, and pamidronate. Off-label BPs therapy was administered in 7 osteoporotic patients. We could point out that the role of non-nitrogen-containing BPs therapy, such as clodronate, in BRONJ development should not be underestimated.

BRONJ was due to oral administration of BPs in 22.66% of patients of this sample and to parenteral administration of BPs in the other 77.34% of patients of this sample. The mean duration of BPs therapy at presentation was 30.2 ± 28.2 months, higher in osteoporotic patients (37 ± 37.2 months) rather than in neoplastic ones (26.3 ± 17.9 months; *t* = 2.8, *P* = 0.0057) (Table 3).

The mean time of BPs therapy suspension before the surgery was 7 ± 7.6 months.

As reported in the literature, the majority of lesions were in Stage 2 AAOMS, because the BRONJ diagnosis is often linked with the appearance of symptoms, which characterize Stage 2.

According to the new dimensional staging, the majority of lesions among neoplastic patients were in Stage III, whereas among osteoporotic patients two-thirds of lesions were equally divided into Stage II and Stage III, requiring major surgery. The medium size of the lesions was 3.8 ± 1.6 cm (range 0.6–8 cm), and the medium lesions number was 1.3 ± 0.6 per patient (range 1–4).

Comorbidity was present in 70.69% (*n* = 41/58) of osteoporotic patients and in 49.65% (*n* = 72/145) of neoplastic patients (*χ* ^2^ = 7.43; *P* = 0.0064). In both neoplastic and osteoporotic patients, there was a higher predilection for mandible involvement rather than maxilla location (mandible-to-maxilla ratio 1.8 : 1), and tooth extraction was the most common triggering factor. The more common symptoms and signs detected were pain and suppuration, followed by paraesthesia, fistulas, and maxillary sinus involvement (Table 4).

The multiple logistic regression model showed a statistically significant association among the dimensional stage III and the duration of BPs exposure (OR = 1.02; *z* = 2.3; *P* = 0.022) and the recurrences (OR = 4.2; *z* = 2.24; *P* = 0.025). These results point out the determinant role of the duration of BPs exposure on the extension of the lesions and the increased odds of recurrences in major lesions. Furthermore, patients with osteoporosis showed the increase of multifocal lesions odds (OR = 1.75; *z* = 11.3; *P* < 0.0001). The result could be related to the lower importance given to this primary disease by both patients and dentists. Patients usually do not report the BPs assumptions for osteoporosis, overlooking their adverse effects, and, on the other hand, dentists do not pay attention to the medical history of the patient.

### 3.2. Clinical Data

The protocol we propose for the management of BRONJ showed optimum results during the follow-up period, which was not less than 12 months in all patients and more than 30 months in 80% of osteoporotic patients.

84.96% of lesions healed, whereas just 12.78% of lesions recurred. Five patients with six lesions succumbed for complications related to their neoplastic disease and chemotherapy (Table 5). Among the thirty-four lesions involving the maxillary sinus and treated by the Caldwell-Luc technique, only 14.7% recurred.

Risks and benefits of continuing BPs therapy should be planned in a multidisciplinary consultation, but, according to the Position Paper of AAOMS, BPs suspension, if systemic conditions permit it, can be indicated even in the early stage of the disease because it could stabilize BRONJ site, reduce the risk of new lesions development, reduce clinical symptoms, and improve postsurgical healing [16]. However, long cessation of BPs therapy can have severe consequences, such as hypercalcemia associated with tumours or an increase of skeletal events in patients affected by metastasis, multiple myeloma, or osteoporosis.

The three cycles of antibiotic association are mandatory remembering the two major theories, “inside-out” and “outside-in” regarding the BRONJ pathophysiology. In the “inside-out” theory, BPs inhibit the osteoclastic activity and suppress the bone turnover, together with the spread of physiologic microdamage and possibly local infection, leading the bone death within the jaw, with subsequent exposure, whereas the “outside-in” theory suggests that a break in the oral mucosa could lead to the ingress of bacteria and local infection which, coupled with poor bone remodelling, leads to bone death. BRONJ may result from a combination of these two mechanisms, and hypovascularity also plays an important role [33, 34].

Both ceftriaxone and metronidazole could cover Gram-positive and Gram-negative bacteria, including anaerobic forms and* Actinomyces*. Particularly, ceftriaxone was preferred for its broad spectrum and relative toxicity, considering the weak defence immune system of the majority of patients with BRONJ, especially neoplastic ones.

The natural bacteria contamination of mouth suggests that a daily careful local irrigation consisting in physiological saline and hydrogen peroxide in the postoperative period is recommended.

The surgical technique, adequate to the dimensional stage and optimised to each patient, is characterized by the bone cortical preservation; thus, it improves the wound healing and implements the reossification, thanks to the scaffold function which is useful also for the gel application made of hyaluronic acid and amino acids.

As reported in the literature, the selective and micrometric cuts of piezosurgery allow the perfect integrity of the osteotomized surfaces with minimal bone loss and induce an earlier increase in BMPs and proteins, controlling the inflammatory process and stimulating the reossification. Moreover, the cavitation effect together with antibiotic therapy seems to be suitable to decrease the microbial aggregation involved in BRONJ process [35].

The intracavitary intraoperative gel filling, followed by application of the same device upon the stitches, is effective in accelerating soft and hard tissues healing, especially in minor defects. In fact, as reported in the literature, it can improve angiogenesis, fibroblast and osteoblast proliferation, collagen biosynthesis, and production of growth factors, as evidenced by MTT test and alkaline phosphatase histochemical staining [36, 37]. In vivo and in vitro studies suggested that hyaluronic acid plays important roles in bone wound healing by enhancement of osteoblast differentiation through the downregulation of BMP-2 antagonists [38, 39], whereas lysine and proline are important metabolic factors regulating collagen matrix synthesis during osteogenesis [40].

This sterile gel formulation of hyaluronic acid and amino acids is a cheap, biocompatible, biodegradable, and useful medical device, able to reset postsurgical morbidity to zero. It shows immediate haemostatic and antioedema effects according to the hygroscopic properties of hyaluronic acid [40]. Furthermore, the gel viscous consistency decreases the bacteria invasion, accelerating the mucosal and bone healing time and the removal of stitches time, even in lesions involving maxillary sinus treated by the Caldwell-Luc technique (Table 6).

Histopathological examination revealed the presence of macro-osteones distant from each other in the lamellar bone, with increased separation of the Haversian canals because of the interosteonic deposition and the newly formed bone with different degrees of calcification. Abundant inflammatory infiltration with large and irregular reabsorption lacunae of the lamellar bone and abundant basophilic bacterial colonies interspersed with necrotic debris were detected.

As reported in the literature, the addition of a nonsurgical laser approach could improve the results of medical and surgical therapy, thanks to the properties of LLLT on the stimulation of reparative process, bone cells proliferation and differentiation, and lymphatic and blood vascularization [32]. The 20 patients treated with a combination of medical, surgical, and biostimulating laser therapies showed the acceleration of mucosal healing time and reossification time, suggesting that LLLT may be a valid technique to support the treatment of BRONJ. The limitation of this technique is that it needs a great cooperation of the patients who have to reach the hospital many times in the first three weeks after surgery for the phototherapy.

Patients cannot resume the BPs therapy until after the surgical area is healed, to reduce the risk of new site development.

## 4. Conclusions

Since the dimensional problem in the resective surgery is important, the new dimensional staging allows us to ensure better patients management considering lesions from a surgical point of view and not from a clinical aspect. The purpose of this staging is to adequate the BRONJ management to each patient choosing between general anaesthesia and conscious sedation, the number of antibiotic cycles, the way of antibiotics administration, the suitable surgical incision, the extension of surgical access, the noble structures involvement, and the adequate wound closure (simple flap, roll flap, or adipose flap), as in oncologic surgery. Furthermore, the impossibility to place bone graft in these patients makes the management worse. Thus, the different surgical approach influences the soft and hard tissues healing time and the postoperative complications (such as oedema, bleeding, wound dehiscence, infections, paraesthesia, and persistent wide bone defects), which become more predictable.

**Figure 3:**
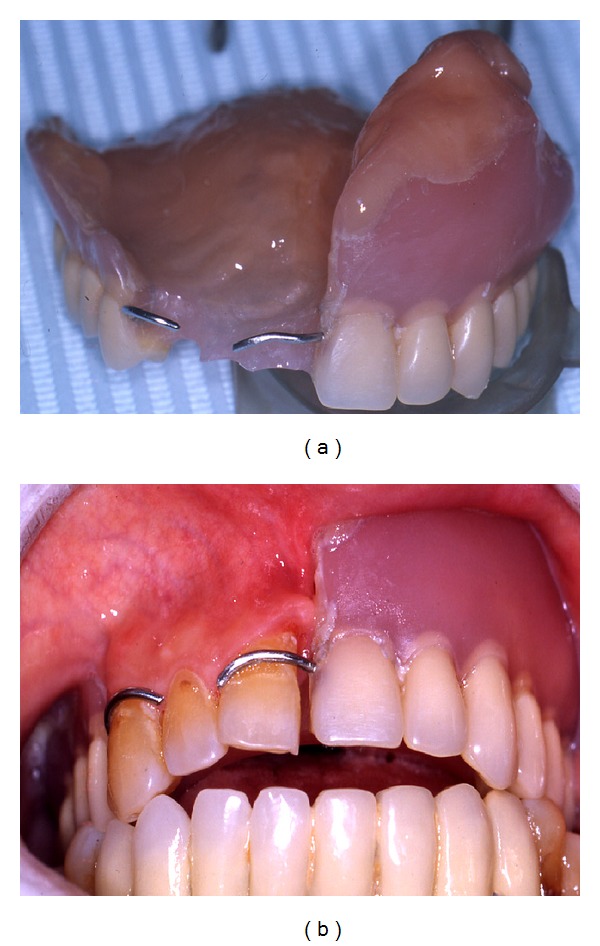
Rehabilitation with social temporary removable prosthesis for aesthetic reasons with good stabilisation of the surgical sites.

**Figure 4:**
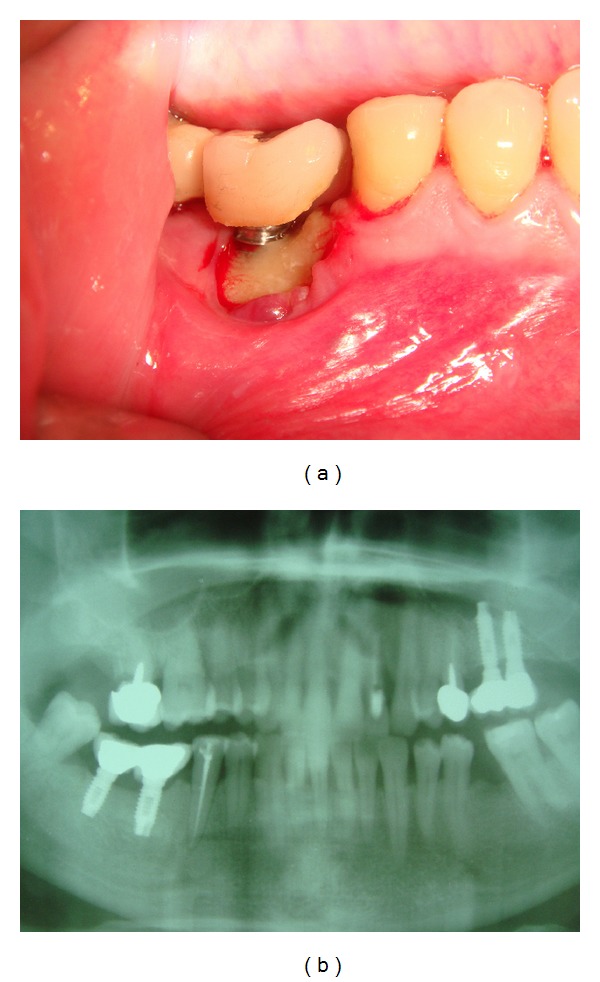
Clinical and radiological aspects of a peri-implantar Stage III BRONJ in a 55-year-old patient with breast cancer.

**Figure 5:**
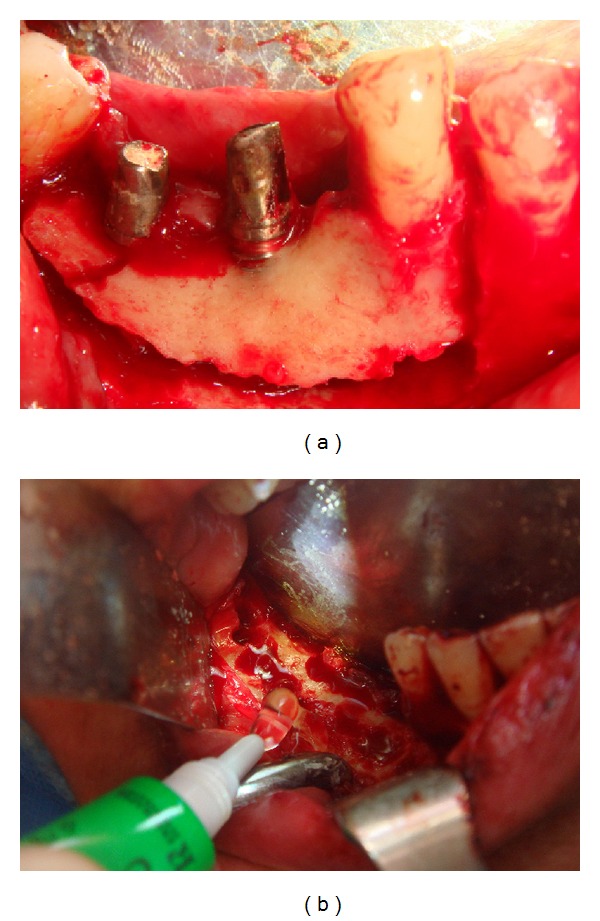
Alveolar bone marginal resection and intraoperative intracavitary application of Aminogam gel.

**Figure 6:**
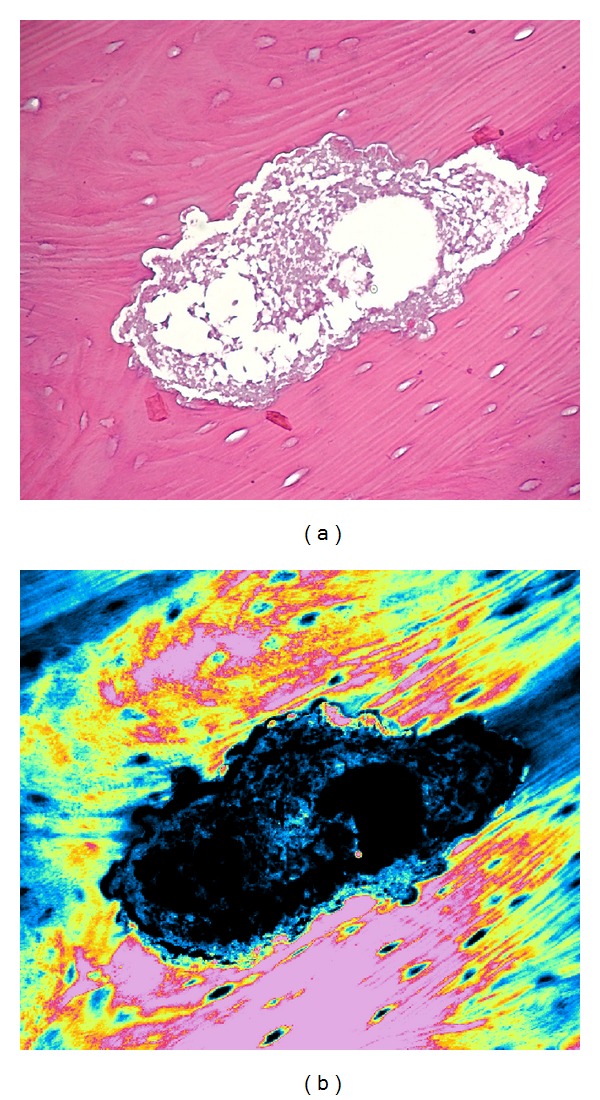
Internal reabsorption of Haversian canals with large and irregular appearance in traditional microscopy (haematoxylin-eosin staining ×100) and the same field in confocal laser scanning microscopy with double laser inducing fluorescence (green and red).

The present study showed the efficacy of the management proposed, which consisted of a combination of BPs therapy suspension, administration of ceftriaxone and metronidazole, surgical debridement or marginal resection according to the stage, hyaluronic acid and amino acids application, and resumption of BPs not before 1 month after surgery, thanks to the high success rate and the good stabilization of the surgical sites observed after a long-term follow-up (Figure 3).

Sterile gel based on hyaluronate and amino acids is a new medical device, biocompatible, extremely cheap, safe, and useful in all surgical procedures in order to obtain faster healing of both hard and soft tissues, without infective complications, thanks to the wound mechanical protection. This could be important especially in BRONJ lesions, which are often prone to difficult, slow, and complicate recovery.

## Figures and Tables

**Table 1:** Summary of different clinical BRONJ staging.

	Marx 2007 [28]	AAOMS 2009 [16]	SICMF and SIPMO 2012 [29]
At-risk category		No apparent exposed/necrotic bone in patients who have been treated with either oral or IV bisphosphonates	

Stage 0	Subclinical damage, microscopically represented by beginner hypocellularity osteoclast apoptosis and decrease of endosteal osteoblast	Nonspecific clinical findings and symptoms such as jaw pain or osteosclerosis but no clinical evidence of exposed bone	

Stage 1	A: painless exposed bone <1 cm B: painless exposed bone >1 cm	Exposed/necrotic bone in patients who are asymptomatic and who have no evidence of infection	Focal BRONJ Clinical signs and symptoms: bone exposure; sudden dental mobility; nonhealing postextraction socket; mucosal fistula; swelling; abscess formation; trismus; gross mandibular deformity; and/or hypoesthesia/paraesthesia of the lips CT finding: increased bone density limited to the alveolar bone region (trabecular thickening and/or focal osteosclerosis), with or without the following signs: markedly thickened and sclerotic lamina dura; persisting alveolar socket; and/or cortical disruption 1a: asymptomatic 1b: symptomatic (pain and purulent discharge)

Stage 2	A: painful and infected single exposed bone <2 cm B: painful and infected single exposed bone >2 cm	Exposed/necrotic bone associated with infection as evidenced by pain and erythema in the region of the exposed bone with or without purulent drainage	Diffuse BRONJ Clinical signs and symptoms: the same as Stage 1 CT findings: increased bone density extended to the basal bone (diffuse osteosclerosis), with or without the following signs: prominence of the inferior alveolar nerve canal; periosteal reaction; sinusitis; sequestra formation; and/or oroantral fistula 1a: asymptomatic 1b: symptomatic (pain and purulent discharge)

Stage 3	A: multiple exposed bone areas without clinical findings of osteolysis, orocutaneous fistula, or pathological fractures B: exposed bone >3 cm or with clinical findings of osteolysis, or orocutaneous fistula, or pathological fractures	Exposed/necrotic bone in patients with pain, infection, and one or more of the following: pathologic fracture, extraoral fistula, or osteolysis extending to the inferior border or sinus floor	Complicated BRONJ The same as Stage 2, with one or more of the following: clinical signs and symptoms: extraoral fistula; displaced mandibular stumps; nasal leakage of fluids CT findings: osteosclerosis of adjacent bones (zygoma, hard palate); pathologic mandibular fracture; and/or osteolysis extending to the sinus floor

**Table 2:** Dimensional staging.

	Clinical and radiological findings	Treatment
Stage 0	No bone exposure with nonspecific radiographic findings, such as osteosclerosis and periosteal Hyperplasia, and nonspecific symptoms, such as pain	Medical therapy and clinical-radiological follow-up
Stage I	Bone exposure and/or radiographic evidences of necrotic bone*, or persisting alveolar sockets <2 cm in the major diameter, with or without pain	Medical therapy, surgical debridement, and LLLT
Stage II	Bone exposure and/or radiographic evidences of necrotic bone* between 2 and 4 cm in the major diameter, with pain responsive to NSAIDs and possible abscesses	Medical therapy and small open-access surgery with piezosurgery of bone margins
Stage III	Bone exposure and/or radiographic evidences of necrotic bone* >4 cm in the major diameter, with strong pain responsive or not to NSAIDs, abscesses, orocutaneous fistula, and/or maxillary sinus and mandibular nerve involvement	Medical therapy and wide open-access surgery with extensive maxillary or mandibular resection, the Caldwell-Luc technique, and piezosurgery of bone margins

*Radiographic evidences of necrotic bone: irregular hyper- and hypocalcified areas and/or bone sequestra.

**Table 3:** Patients clinical data (*N* = 203).

	*N*	%
Patients characteristics		
Males	50	24.63%
Females	153	75.37%
Mean age	67.8 ± 11.3	
Neoplastic patients	145	71.43%
Breast cancer	58	40%
Multiple myeloma	42	28.97%
Prostate cancer	20	13.79%
Lung cancer	5	3.45%
Others	20	13.79%
Osteoporotic patients	58	28.57%

Type of BPs treatment		
Oral administration	46	22.66%
Parenteral administration	157	77.34%
Neoplastic patients		
Zoledronic acid	137	94.48%
Clodronate	4	2.76%
Risedronate	3	2.07%
Pamidronate	1	0.7%
Mean duration therapy	26.3 ± 17.9 months	
Osteoporotic patients		
Alendronate	30	51.72%
Clodronate	8	13.79%
Ibandronate	5	8.62%
Zoledronic acid	4	6.9%
Risedronate	4	6.9%
Off-label therapy	7	12.1%
Mean duration therapy	37 ± 37.2 months	

**Table 4:** BRONJ lesions (*N* = 277).

	*N*	%
*Clinical stage (AAOMS) *		
Lesions in neoplastic patients	195	73.3%
Stage 0	1	0.51%
Stage 1	14	7.18%
Stage 2	115	58.97%
Stage 3	65	33.33%
Lesions in osteoporotic patients	71	26.7%
Stage 0	1	1.4%
Stage 1	2	2.82%
Stage 2	53	74.65%
Stage 3	15	21.13%
*Dimensional stage *		
Lesions in neoplastic patients	195	73.3%
Stage 0	1	0.51%
Stage I	22	11.28%
Stage II	58	29.74%
Stage III	114	58.5%
Lesions in osteoporotic patients	71	26.7%
Stage 0	1	1.41%
Stage I	13	18.31%
Stage II	28	39.44%
Stage III	29	40.84%
Medium size	3.8 ± 1.6 cm	
History of extractions	169	63.53%
Initial symptoms per lesion (*N* = 266)		
Pain	233	87.59%
Suppuration	198	74.43%
Paraesthesia	78	29.32%
Fistulas	46	17.29%
Maxillary sinus involvement	34	12.78%

**Table 5:** Treatment outcomes (*N* = 266).

	*N*	%
*Clinical success *	226	84.96%
Neoplastic patients (195 lesions)	159	81.54%
Osteoporotic patients (71 lesions)	67	94.37%
*Recurrences *	34	12.78%
Neoplastic patients (195 lesions)	30	15.39%
Osteoporotic patients (71 lesions)	4	5.63%
*Lesions in patients who succumbed *		
Neoplastic patients (195 lesions)	6	3.08%

**Table 6:** Soft tissues healing time (days).

	Stitches removal	Complete wound healing
Stage I	7–9	9–12
Stage II	12–15	14–21
Stage III	15–21	25–28
The Caldwell-Luc technique	20–23	40–45
